# Machine Learning and Novel Biomarkers Associated with Immune Infiltration for the Diagnosis of Esophageal Squamous Cell Carcinoma

**DOI:** 10.1155/2022/6732780

**Published:** 2022-08-30

**Authors:** Jipeng Zhang, Nian Zhang, Xin Yang, Xiangbin Xin, Cheng-hui Jia, Sen Li, Qiang Lu, Tao Jiang, Tao Wang

**Affiliations:** ^1^Department of Thoracic Surgery, Tangdu Hospital, The Air Force Military Medical University, Xi'an 710038, Shaanxi, China; ^2^Department of Anesthesiology, Tangdu Hospital, The Air Force Military Medical University, Xi'an 710038, Shaanxi, China; ^3^Pathology Department, The Second Affiliated Hospital of Shaan Xi University of Traditional Chinese Medicine, Xi'an 710038, Shaanxi, China; ^4^Department of Thoracic Surgery, The First Affiliated Hospital of Xi'an Medical College, Xi'an 710000, China; ^5^Department of Cardio-Thoracic Surgery, Luohe Centre Hospital, Luohe 462000, Henan, China

## Abstract

Esophageal squamous cell carcinoma (ESCC) accounts for the main esophageal cancer type, which is related to advanced stage and poor survivals. Therefore, novel diagnostic biomarkers are critically needed. In the current research, we aimed to screen novel diagnostic biomarkers based on machine learning. The expression profiles were obtained from GEO datasets (GSE20347, GSE38129, and GSE75241) and TCGA datasets. Differentially expressed genes (DEGs) were screened between 47 ESCC and 47 nontumor samples. The LASSO regression model and SVM-RFE analysis were carried out for the identification of potential markers. ROC analysis was carried out to assess discriminatory abilities. The expressions and diagnostic values of the candidates in ESCC were demonstrated in the GSE75241 datasets and TCGA datasets. We also explore the correlations between the critical genes and cancer immune infiltrates using CIBERSORT. In this study, we identified 27 DEGs in ESCC: 5 genes were significantly elevated, and 22 genes were significantly decreased. Based on the results of the SVM-RFE and LASSO regression model, we identified five potential diagnostic biomarkers for ESCC, including GPX3, COL11A1, EREG, MMP1, and MMP12. However, the diagnostic values of only GPX3, MMP1, and MMP12 were confirmed in GSE75241 datasets. Moreover, in TCGA datasets, we further confirmed that GPX3 expression was distinctly decreased in ESCC specimens, while the expression of MMP1 and MMP12 was noticeably increased in ESCC specimens. Immune cell infiltration analysis revealed that the expression of GPX3, MMP1, and MMP12 was associated with several immune, such as T cells CD8, macrophages M2, macrophages M0, and dendritic cells activated. Overall, our findings suggested GPX3, MMP1, and MMP12 as novel diagnostic marker and correlated with immune infiltrates in ESCC patients.

## 1. Introduction

Esophageal cancer (EC) is one of the most common malignancies worldwide, which is always accompanied by high morbidity and mortality [1]. Esophageal cell squamous carcinoma (ESCC) accounts for over 80% of all cases of EC in China [2]. Surgery, radiation therapy, and chemotherapy are the only treatment options that are currently available; despite the significant progress that has been made in the treatment of this illness, the patient survival rate within five years is still extremely low [3, 4]. This is due to the fact that the only treatment options that are currently available are those three. Metastatic ESCC patients have a five-year survival rate of fewer than 5% [5]. ESCC often spreads to the liver, lung, bone, and brain [6]. For the detection of ESCC, they are ineffective because they lack appropriate sensitivity and specificity [7]. Therefore, novel and reliable molecular biomarkers to complement and improve on current ESCC screening strategies are urgently needed.

The investigation of gene expression profiles using microarrays has become a frequent method for locating important hub genes and important pathways [8]. In this day and age of integrated bioinformatics, it is not a problem to get data; rather, the task of normalization appears to be a challenging one. It is possible to perform prognosis studies on cancer patients using microarray techniques in addition to identifying genes associated with various diseases and potential antitumor medication targets [9, 10]. In addition, microarray techniques have an important ability in analyzing the associations between the expression of functional genes and their modulation [11, 12]. In the field of clinical research, they are also responsible for contributing ideas for the diagnosis and treatment of specific disorders. We found that there have been studies exploring the diagnostic genes of many types of tumors, but the application of machine-learning for the identification of novel diagnostic biomarkers for ESCC was rarely reported. In the current study, we performed a joint analysis in multidatabases to explore diagnostic marker genes for ESCC patients.

## 2. Materials and Methods

### 2.1. Microarray Data

The microarray dataset GSE20347, GSE38129, and GSE75241 was downloaded from the Gene Expression Omnibus (GEO) database. The GSE20347 dataset included 17 pairs of ESCC and nontumor specimens, whereas the GSE38129 dataset included 30 pairs of ESCC and nontumor specimens. Due to the fact that the GSE38129 datasets share a platform and are important for merging data from a variety of datasets, they have been combined into a metadata cohort for the purpose of doing additional integration analysis. In addition, the combat function contained inside the “SVA” software package in R was utilized in order to eliminate the batch effect. In addition, the validation cohort comprised 15 pairs of ESCC tissues and neighboring normal tissues, which were taken from the GSE75241 datasets.

### 2.2. Identification of Differentially Expressed Genes (DEGs)

The DEG analysis was carried out with the help of the Limma program [13]. In order to evaluate the changes in gene expression, an empirical Bayesian methodology was adopted, and moderated *t*-tests were utilized. The DEGs are genes that had an adjusted *p* value that was lower than 0.05 and had an absolute fold change that was higher than 3.

### 2.3. GO Term and KEGG Pathway Enrichment Analysis

The biological importance of DEGs was investigated using GO term enrichment analysis, which included biological processes, cellular components, and molecular functions. This research was conducted using the “GOstats” program included in Bioconductor. The KEGG pathway enrichment analysis of DEGs was carried out by the “GeneAnswers” Bioconductor program in order to identify important pathways that are closely associated with the beginning and development of ESCC. In order to reach statistical significance and achieve significant enrichment, a *p* value of less than 0.05 was required.

### 2.4. Novel Diagnostic Biomarkers Screening

When doing five-fold cross-validation, a technique known as least absolute shrinkage and selector operation (LASSO) and support vector machine-recursive feature elimination (SVM-RFE) were employed, respectively, to filter the critical genes [14, 15]. Then, in order to filter the essential diagnostic genes, we pooled the results that the LASSO and SVM-RFE algorithms had produced. The genes that were shared by the two methods were incorporated, and the expressions of novel genes were checked for accuracy using the GSE75241 datasets.

### 2.5. Diagnostic Value of Critical Genes in ESCC

In order to determine whether or not the found biomarkers had any predictive power, we constructed a ROC curve by comparing the levels of mRNA expression in 47 ESCC tumor specimens to 47 nontumor tissues. The value of AUC was used to measure the diagnostic efficiency in distinguishing ESCC specimens from nontumor specimens, and this finding was then verified using the GSE75241 dataset.

### 2.6. Estimation of Immune Cell Abundance

Based on the reference signature matrix of 547 genes, we employed CIBERSORT to analyze the percentages of various immune cells in tumor and nontumor specimens. When we ran the program with the default LM22 feature matrix at 1000 permutations, we submitted the data of gene expressions generated from the sample mixture file to the CIBERSORT web page (https://cibersort.stanford.edu/). A mixture sample's relative immune cell fraction was estimated using CIBERSORT and can be applied to compare immune cell populations within and across studies.

### 2.7. Statistical Analysis

All statistical analyses were conducted using R (version 3.6.3, R Core Team, Massachusetts, USA). *p* < 0.05 was considered statistically significant.

## 3. Results

### 3.1. Identification of DEGs in ESCC

**Figure 1:**
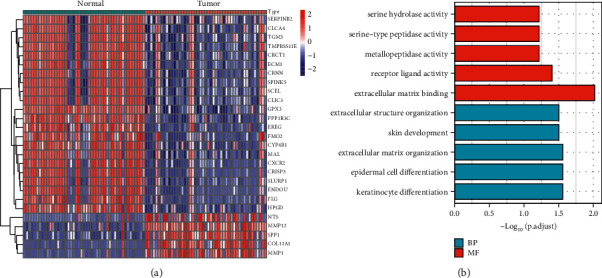
Identification of DEGs in ESCC and their enrichment analysis. (a) A total of 139 DEGs were obtained between ESCC specimens and nontumor specimens, which were shown in the heat map. (b) Representative results of GO analyses in TCGA.

Extensive retrospective analysis was performed on the GSE20347 and GSE38129 GEO datasets, which contained data on 47 ESCC and 47 nontumor samples. We used the Limma package to remove batch effects before analyzing the metadata DEGs. There were a total of 27 DEGs collected: 5 genes were significantly elevated, and 22 genes were significantly decreased (Figure 1(a)).

### 3.2. GO and KEGG Pathway Enrichment Analyses of DEGs

Later, we carried out GO assays using the “clusterProfiler” R package and observed that, in the BP group, the DEGs were mainly involved in extracellular structure organization, skin development, extracellular matrix organization, epidermal cell differentiation, and keratinocyte differentiation. In the MF group, the DEGs were mainly involved in serine hydrolase activity, serine-type peptidase activity, metallopeptidase activity, receptor ligand activity, and extracellular matrix binding (Figure 1(b)). However, the results of KEGG did not show any significant terms.

### 3.3. Identification and Validation of Diagnostic Feature Biomarkers

**Figure 2:**
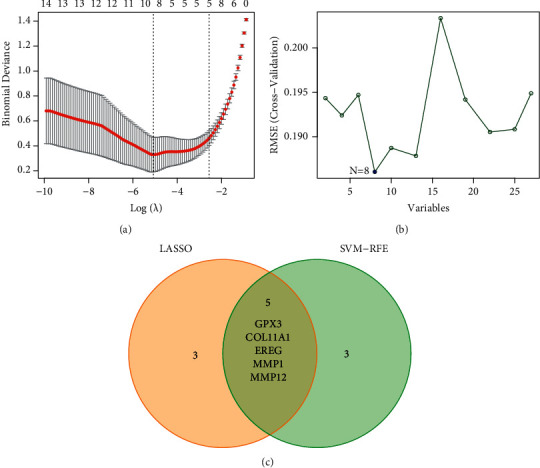
Identification of novel diagnostic biomarkers for ESCC diagnosis. (a) Tuning feature selection in the LASSO. (b) A plot of markers selection via the SVM-RFE algorithm. (c) Venn diagram demonstrating five diagnostic markers shared by the LASSO and SVM-RFE algorithms.

**Figure 3:**
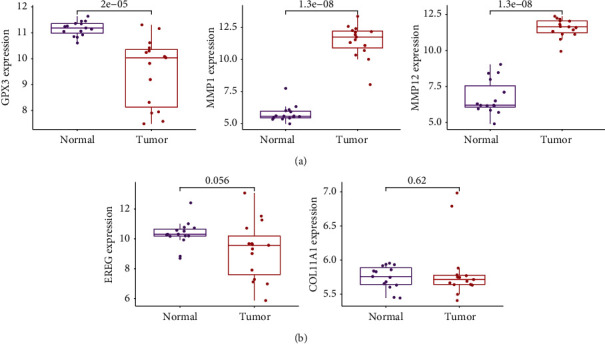
The expression of GPX3, COL11A1, EREG, MMP1, and MMP12 in ESCC specimens and nontumor from GSE75241 datasets.

Researchers made use of two distinct algorithms in their search for possible biomarkers. Regression analysis carried out with the LASSO algorithm helped reduce the number of DEGs, which led to the identification of eight biomarkers for ESCC (Figure 2(a)). The SVM-RFE algorithm was used to narrow down the features of the DEGs to a selection of five characteristics (Figure 2(b)). In the end, the four traits that were found to overlap between these two methods, known as GPX3, COL11A1, EREG, MMP1, and MMP12, were chosen (Figure 2(c)). GSE75241 dataset was utilized to check the expressions of five characteristics to obtain more accurate and reliable results. ESCC tissue had significantly higher levels of GPX3, MMP1, and MMP12 expression than normal tissues (Figure 3(a)). However, regarding the levels of expression of COL11A1 and EREG, there was not a discernible difference between the two groups (Figure 3(b)).

### 3.4. Diagnostic Effectiveness of Novel Biomarkers in ESCC

**Figure 4:**
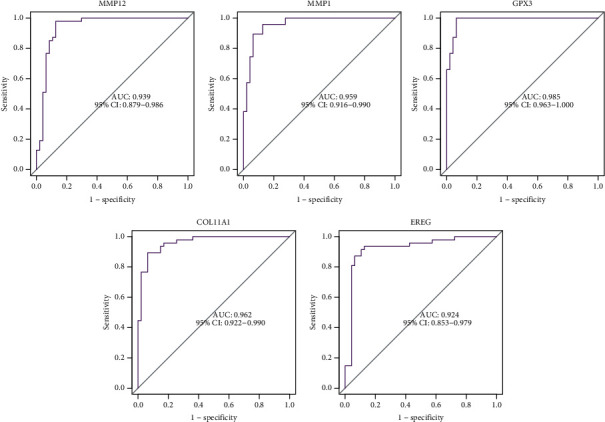
ROC curve of the five diagnostic markers using GSE20347 and GSE38129 datasets.

**Figure 5:**
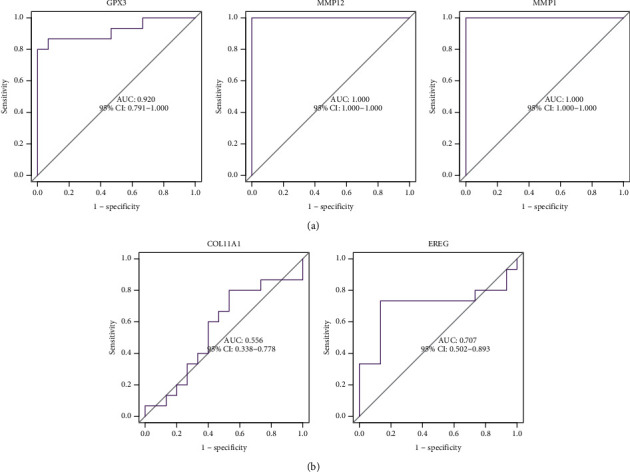
ROC curve of the five diagnostic markers using GSE75241 datasets.

The diagnostic abilities of GPX3, MMP1, and MMP12 in discriminating ESCC from nontumor specimens confirmed excellent diagnostic values, with an AUC of 0.939 (95% CI 0.879–0.986) in MMP12, AUC of 0.959 (95% CI 0.916–0.990) in MMP1, AUC of 0.985 (95% CI 0.963–0.100) in GPX3, AUC of 0.962 (95% CI 0.922–0.990), and AUC of 0.924 (95% CI 0.853–0.979) (Figure 4). Moreover, a powerful discrimination ability was demonstrated in the GSE75241 dataset with an AUC of 0.920 (95% CI 0.791–0.100) in GPX3, AUC of 1.000 (95% CI 1.000–1.000) in MMP12, and AUC of 1.000 (95% CI 1.000–1.000) in MMP1 (Figure 5(a)). However, the AUC for COL11A1 and EREG was 0.556 and 0.707 (Figure 5(b)).

### 3.5. Pan‐Cancer Expression Landscape of GPX3, MMP1, and MMP12 Based on TCGA Datasets

**Figure 6:**
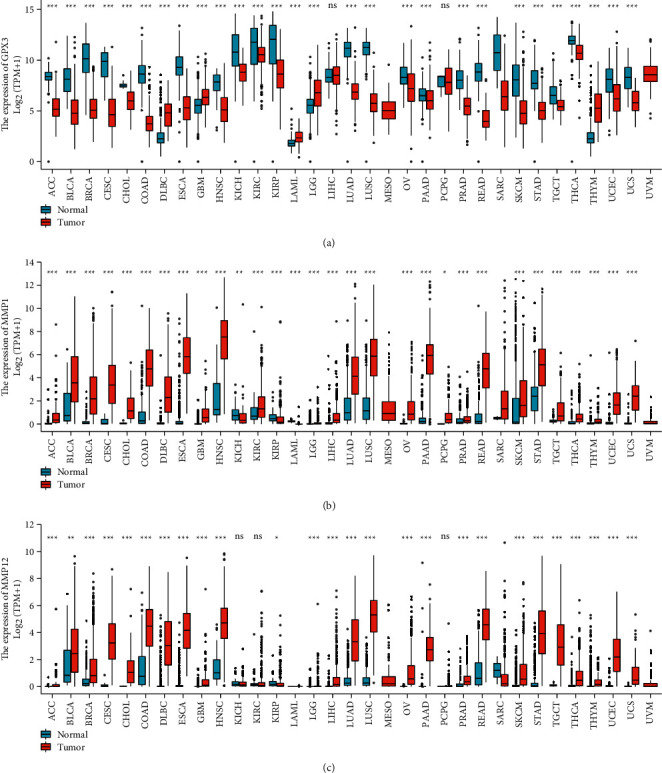
The pan-cancer analysis using TCGA datasets.

**Figure 7:**
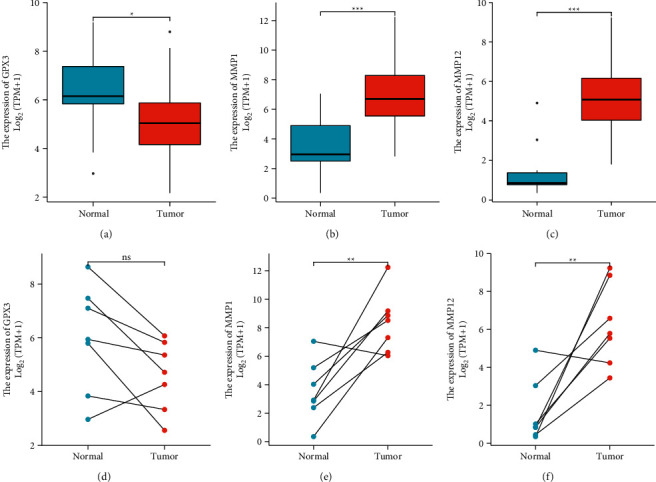
The expression of GPX3, MMP1, and MMP12 between ESCC specimens and nontumor specimens from TCGA datasets using (a–c) unpaired *t*-test and (d, e) paired *t*-test. ^*∗∗∗*^*p* < 0.001, ^*∗∗*^*p* < 0.01, and ^*∗*^*p* < 0.05.

We conducted pan-cancer assays based on TCGA datasets to investigate the putative roles of GPX3, MMP1, and MMP12 in malignancies. According to our findings, the GPX3 expression is markedly decreased in most cancers (Figure 6(a)), while MMP1 and MMP12 expression was distinctly upregulated in most types of tumors (Figures 6(b) and 6(c)). According to our findings in Figure 7(a), we found that MMP1 and MMP12 expression was markedly elevated in ESCC samples compared to nontumor samples, while the GPX3 expression was decreased (Figures 7(b) and 7(c)). However, the results of paired *t*-test did not show a distinct difference of GPX3 expressions between ESCC samples and nontumor samples (Figure 7(d)), while the MMP1 and MMP12 expression was also further confirmed by the use of paired *t*-test (Figures 7(e) and 7(f)).

### 3.6. Correlation of GPX3, MMP1, and MMP12 Expression with Tumor-Infiltrating Immune Cells (TICs)

**Figure 8:**
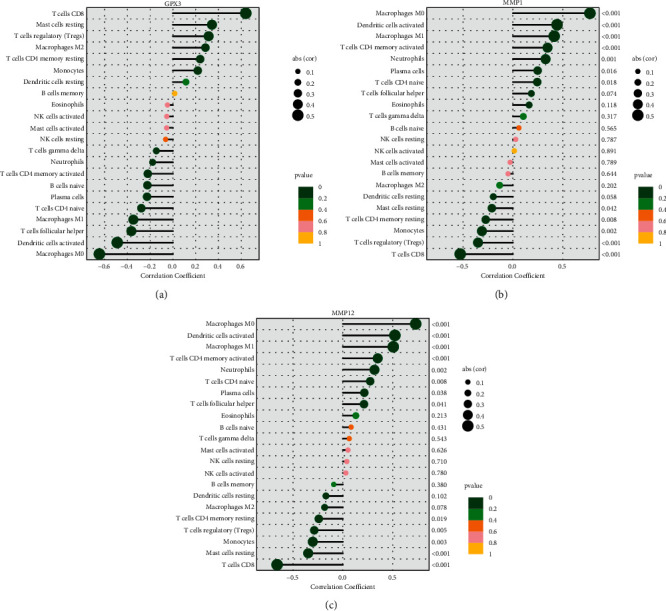
Relationships between (a) GPX3, (b) MMP1, (c) MMP12, and infiltrating immune cells in ESCC.

The CIBERSORT tool in R programming language was used to conduct additional studies to verify the association between GPX3, MMP1, and MMP12 expression and the TME.  Figure S1(a) shows the establishment of 22 types of immune cell profile in ESCC samples and nontumor samples, and the relationship between these TICs was exhibited by the use of heatmap (Figure S1(b)). Subsequently, we compared the proportions of TICs in the ESCC samples with those in the nontumor samples, and we found that differences in B cells naïve, plasma cells, T cells CD8, T cells CD4 naïve, T cells CD4 memory resting, T cells CD4 memory activated, T cells follicular helper, T cells regulatory (Tregs), monocytes, macrophages M0, macrophages M1, macrophages M2, dendritic cells activated, mast cells resting, and neutrophils were statistically significant (Figure S1(c)). Moreover, we observed that the GPX3 expression was associated with the expression of T cells CD8, mast cells resting, T cells regulatory (Tregs), macrophages M2, T cells CD4 memory resting, monocytes, dendritic cells resting, T cells gamma delta, neutrophils, T cells CD4 memory activated, B cells naïve, plasma cells, T cells CD4 naïve, macrophages M1, T cells follicular helper, dendritic cells activated, and macrophages M0 (Figure 8(a)). MMP1 expression was associated with the expression of macrophages M0, dendritic cells activated, macrophages M1, T cells CD4 memory activated, neutrophils, plasma cells, T cells CD4 naïve, mast cells resting, T cells CD4 memory resting, monocytes, T cells regulatory (Tregs), and T cells CD8 (Figure 8(b)). MMP12 expressions were related to the expressions of macrophages M0, dendritic cells activated, macrophages M1, T cells CD4 memory activated, neutrophils, T cells CD4 naïve, plasma cells, T cells follicular helper, T cells CD4 memory resting, T cells regulatory (Tregs), monocytes, mast cells resting, and T cells CD8 (Figure 8(c)).

## 4. Discussion

The most common kind of esophageal cancer found all over the world is ESCC [16]. ESCC ranks as the fourth greatest cause of death from cancer-associated causes in China [17]. Even with the recent advances that have been made in diagnosis and therapy, the outlook for ESCC remains dismal [18, 19]. Patients diagnosed with ESCC have a survival rate of fewer than 30 percent after 5 years. Despite the fact that various biomarkers for ESCC have been discovered, the therapeutic relevance of the majority of them has not been validated [20, 21]. Therefore, there is an immediate need for research into more effective biomarkers for the diagnosis of ESCC.

To the best of our knowledge, this is the first retrospective study that has used GEO databases to look for diagnostic indicators that are associated with immune cell infiltration in patients with ESCC. Two cohorts were drawn from the GEO datasets, and the data were subjected to an integrated analysis. There were 27 DEGs in total: five genes showed distinct increases, whereas 22 genes showed distinct decreases. The results of enrichment analyses revealed that the dysregulated genes were primarily involved in matrix organization, skin development, extracellular, extracellular structure organization, epidermal cell differentiation, and keratinocyte differentiation. In the MF group, the abnormal expressed genes were primarily involved in serine hydrolase activity, serine-type peptidase activity, metallopeptidase activity, receptor ligand activity, and extracellular matrix binding. A total of five diagnostic indicators have been discovered using two machine-learning algorithms, including the following: GPX3, COL11A1, EREG, MMP1, and MMP12. In addition, we used GSE75241 datasets to further demonstrate our findings, and the diagnostic value of GPX3, MMP1, and MMP12 was further confirmed.

The human matrix metalloproteinases (MMPs) family belongs to the metzincin superfamily [22]. Extracellular matrix degradation is aided by MMPs, which catalyze proteolytic processes [23]. Several types of cancers are affected by MMPs in different ways [24, 25]. As of this writing, a total of 24 MMPs have been discovered (MMP1, 2, 3, 4, 5, 7, 8, 9, 10, 11, 12, 13, 14, 15, 16, 17, 18, 19, 20, 21, 23, 23a/23b, 24, 25, 26, 27, and 28). Liu et al. reported that lymph node metastases, microvessel density, and an advanced TNM stage were all linked to ESCC patients with elevated MMP1 expression. Multivariate and Kaplan–Meier analyses found that MMP1 was a significant independent predictor of overall survival of ESCC patients. In vitro experiments showed that MMP1 overexpression improved cell viability, colony formation, and cell movement capacities. The opposite effect was observed when MMP1 was knocked down in ESCC cells. The PI3K/AKT pathway was activated when MMP1 was expressed ectopically in tumor cells, resulting in tumor development and metastasis [26]. In addition, the prognostic values of MMP12 have been reported in several previous studies [27–29]. In this study, according to the results of GSE20347, GSE38129, GSE75241, and TCGA datasets, we further confirmed that MMP1 and MMP12 expressions were distinctly increased in ESCC specimens compared with nontumor samples. ROC assays also confirmed their diagnostic value in screening ESCC samples from normal samples. Overall, the results from machine-learning, together with previous findings, suggested MMP1 and MMP12 as critical diagnostic and prognostic factors for ESCC. However, research on the role of MMP1 and MMP12 in ESCC progression needed to be conducted in both vitro and animal models.

Glutathione peroxidase (GPX) is an important peroxide that has been demonstrated to be widely involved in nontoxic compounds, the reduction of toxic peroxides into hydroxyl compounds, and the decomposing of enzymes [30, 31]. Growing studies have confirmed that GPX reduces the occurrence and development of tumors [32, 33]. It has been found that the methylation of GPX3, a member of the GPX family of tumor-suppressor genes, increases the risk of breast, live, and cervical cancer substantially [34–36]. In ESCC, GPX3 has been reported to be lowly expressed in ESCC and its overexpression promoted the migration and invasion of ESCC cells via regulating FAK/AKT pathway [37]. Our findings were consistent with previous findings.

The role of the tumor microenvironment (TME) in the development of the tumor was proven by an increasing amount of evidence [38]. The malignant characteristics of cancer, such as immortal proliferation, resistance to apoptosis, and evasion of immune surveillance, are thought to be at least partially caused by cooperative interactions between cancer cells and the cells that sustain them [39, 40]. As a result, the TME exerts a considerable amount of impact over the therapeutic response and clinical outcome in cancer patients. Thus, we evaluated the correlation between GPX3, MMP1, and MMP12 expressions and immune cell infiltration in ESCC. Interesting, we found that the expression of GPX3, MMP1, and MMP12 was distinctly associated with the expressions of many immune cells. Therefore, the positive correlation between the amounts of several immune cells and the expressions of GPX3, MMP1, and MMP12 in ESCC patients suggested that GPX3, MMP1, and MMP12 were responsible for the maintenance of an immune-active condition in TME.

However, our present study has some limitations. Firstly, considering the limited size of the sample, it will be necessary to do extensive clinical tests. Secondly, we fail to evaluate the expression profile of GPX3, MMP1, and MMP12 in the serum/plasma samples in patients with ESCC. Analyzing the biomarkers present in the serum and plasma samples could be an effective way to evaluate the response to treatment in real time. Additionally, the function of GPX3, MMP1, and MMP12 remained largely unclear, and their function and mechanism were worth further exploration by molecular function experiment. We plan to better incorporate more data sets to demonstrate our findings in the next paper. In order to make these more accurate, we intend to obtain tumor specimens in addition to clinical data and demonstrate the accuracy of the results via tests.

## 5. Conclusion

We identified GPX3, MMP1, and MMP12 as novel diagnostic genes for ESCC. Our research also provided methods to evaluate those that had a higher potential to benefit from immunotherapy and identified a number of candidate therapeutic targets that could provide a more efficient form of treatments.

## Data Availability

The data used to support the findings of this study are included within the article. Additional data can be made available from the corresponding author upon request. Disclosure Jipeng Zhang, Nian Zhang, and Xin Yang should be considered as equal co-first authors

## Supplementary Materials

Supplementary Materials
*Figure S1.* (a, b) The proportion of the 22 immune cells detected by the CIBERSORT algorithm. (c) The differences in the structure of TIICs between normal tissue and ESCC tissues.
