# Supplementary figures and images for: Machine Learning and Novel Biomarkers Associated with Immune Infiltration for the Diagnosis of Esophageal Squamous Cell Carcinoma
Source: J Oncol. 2022 Aug 30;2022:6732780. doi: 10.1155/2022/6732780 (PMC9448540; doi:10.1155/2022/6732780)

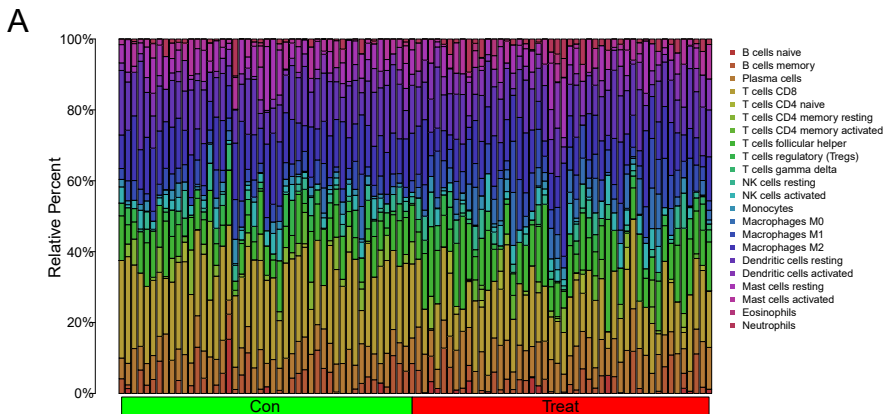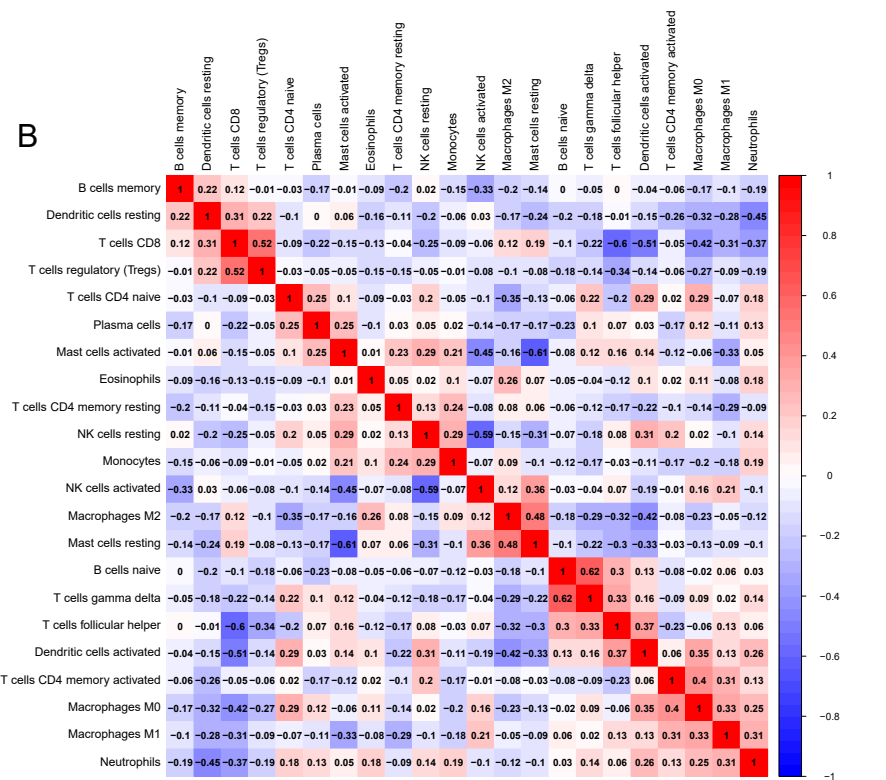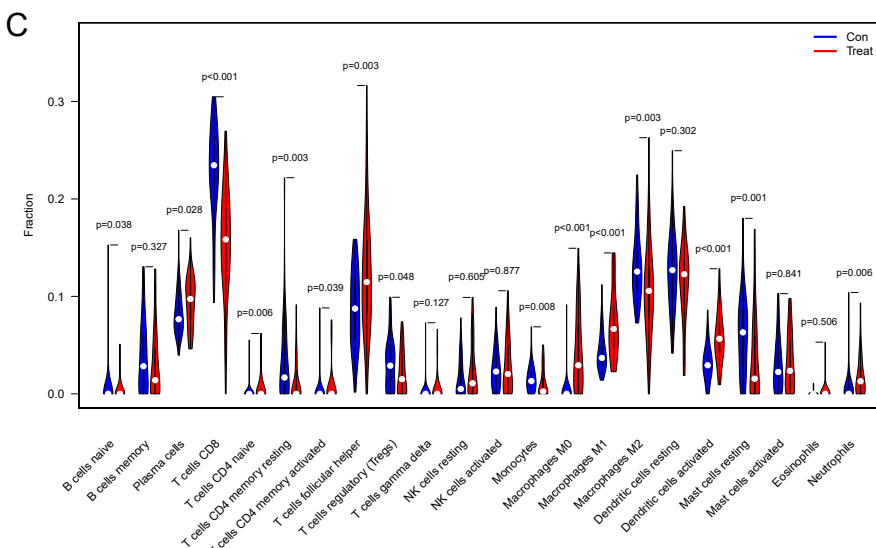

Supplement: Supplementary Materials — Figure S1. (a, b) The proportion of the 22 immune cells detected by the CIBERSORT algorithm. (c) The differences in the structure of TIICs between normal tissue and ESCC tissues. [file 6732780.f1.pdf]
